# Incidence of hospitalizations related to Lyme disease and other tick-borne diseases using Discharge Abstract Database, Canada, 2009−2021

**DOI:** 10.1371/journal.pone.0312703

**Published:** 2024-10-25

**Authors:** Salima Gasmi, Nicholas H. Ogden, Annie-Claude Bourgeois, Maria Elizabeth Mitri, Peter Buck, Jules K. Koffi

**Affiliations:** 1 Centre for Food-borne, Environmental and Zoonotic Infectious Diseases, Public Health Agency of Canada, Saint-Hyacinthe, Québec, Canada; 2 Groupe de Recherche en Épidémiologie des Zoonoses et Santé Publique, Université de Montréal, St-Hyacinthe, Québec, Canada; 3 Public Health Risk Sciences Division, Scientific Operations and Response, National Microbiology Laboratory, Public Health Agency of Canada, Saint-Hyacinthe, Québec, Canada; 4 Centre de Recherche en Santé Publique, Université de Montréal, Montréal, Québec, Canada; 5 Centre for Food-borne, Environmental and Zoonotic Infectious Diseases, Public Health Agency of Canada, Ottawa, Ontario, Canada; Kerman University of Medical Sciences, ISLAMIC REPUBLIC OF IRAN

## Abstract

To estimate rates of hospitalizations for tick-borne diseases (TBDs) in Canada, retrospective analysis was conducted to determine the incidence of patients diagnosed with TBDs during their hospital stay in Canada, and describe demographic characteristics, temporal trends and geographic distributions, from 2009 through 2021. Codes from the International Classification of Diseases, Tenth Revision (ICD-10-CA) were used to capture diagnoses of TBDs in the Discharge Abstract Database (DAD) in Canadian hospitals. From 2009 through 2021, 1,626 patients were diagnosed with TBDs during their hospital stay. Of these, 1,457 were diagnosed with Lyme disease (LD), 162 with other TBDs, and seven were diagnosed with more than one TBD. Annual hospitalization counts for LD showed a significant increase from 50 in 2009 to 259 in 2021 (incidence rate per 100,000 population of 0.1 and 0.7, respectively). Epidemiologic patterns for hospitalized LD cases, including increases and variation in annual incidences, seasonality, demographics and geographic distribution, are consistent with those elucidated in national LD surveillance data. Amongst 162 patients diagnosed with other tick-borne diseases, discharge diagnoses were: rickettsiosis (32.7%), spotted fever due to *rickettsia rickettsii* (23.5%), tularemia (21.0%), babesiosis (8.6%), other tick-borne viral encephalitis (6.2%), tick-borne relapsing fever (4.9%), and Colorado tick fever (0.6%). Annual incidence increased only for rickettsiosis from 3 to 12 patients over the study period. Monitoring the data of hospitalizations using the DAD provided insights into the burden of emerging TBDs, the severity of illnesses and the population most at risk.

## Introduction

Ticks can transmit a variety of pathogens that cause disease in humans and animals and may carry more than one pathogen at a time. The emergence of tick-borne diseases (TBDs) in Canada, which is now a significant public health issue, is driven in part by climate change which is leading to increasingly suitable climatic and environmental conditions for tick survival and reproduction, and range expansion of vector ticks [1].

In North America, the black-legged tick, *Ixodes scapularis*, the primary vector of the bacterial agent of Lyme disease *Borrelia burgdorferi* sensu stricto, has expanded its range north into southeastern and southcentral Canada. As well as *B*. *burgdorferi*, other pathogens transmitted by *I*. *scapularis*, including *Anaplasma phagocytophilum*, *Babesia microti*, Powassan virus, *Borrelia miyamotoi* and *Borrelia mayonii* are emerging in Canada, with most also transmitted by *I*. *pacificus* ticks in British Columbia [1–5]. Populations of other endemic tick species that may transmit some of these pathogens are also expanding [2]. Other tick transmitted pathogens are endemic to Canada, including *Borrelia hermsii* (the agent of tick-borne relapsing fever transmitted by soft-bodied ticks) and Colorado tick fever in British Columbia [6] and the Canadian Prairie provinces [7], and *Rickettsia rickettsii* (the agent of Rocky Mountain spotted fever; RMSF) in southern areas of the Central provinces [1].

Lyme disease and tularemia are nationally notifiable diseases and for reported cases, the Canadian notifiable disease surveillance system (CNDSS) collects demographic information, episode date and case classification from the provinces and territories. The Lyme Disease Enhanced Surveillance (LDES) system captures additional data on LD cases, including possible geographic location of exposure, clinical manifestations, and results of laboratory testing. Despite the valuable information provided by these routine surveillance systems, lack of information on hospitalization nationwide limits the ability to estimate the burden of TBDs including LD-related hospitalizations.

The Canadian Institute for Health Information (CIHI) provides standardized data and information on hospitalized patients in Canada through the Discharge Abstract Database (DAD). The administrative and clinical information recorded in the DAD and the International Classification of Diseases, Tenth Revision (ICD-10-CA) codes have been used in several studies and validated for carrying out surveillance in Canada [8–10].

This study aims to use the DAD information to determine the incidence of patients diagnosed with TBDs during their hospital stay in Canada, describe their characteristics, and determine temporal trends and geographic distribution, from 2009 through 2021.

## Methods

The data used in this study encompass administrative, clinical and demographic information captured in the DAD from Canadian acute care facilities. The data cover the period of 2009−2021 and have been provided to CIHI by participating provinces and territories since 2006, when physicians and specialists were required to use the ICD-10-CA coding system in Canada. The data used is housed at the Public Health Agency of Canada, organized (with personal identifiers removed) under strict confidentiality CIHI’s guidelines. As a result, no ethics board approval was required. The data access date was April 3, 2023.

A literature review was conducted to create a list of TBDs for inclusion in this analysis. The selection of TBDs was based on reported human infections in Canada [4, 6, 11–13], the presence of TBD-associated pathogens of public health importance in ticks collected in Canada [14–18], and the growing incidence of TBDs in neighbouring states of the USA from which migratory birds and terrestrial hosts could carry ticks that harbor these pathogens into Canada [2, 19, 20].

The ICD-10-CA disease codes were identified for the selected TBDs including LD, babesiosis, Colorado tick fever, tick-borne relapsing fever, spotted fever (including RMSF), and tularemia (recognising that tularemia may be acquired from a range of transmission routes other than tick-borne; [21]). As no specific codes for Powassan virus disease and anaplasmosis were available in the ICD-10-CA code list, broader more generic codes were used in this analysis including "Other tick-borne viral encephalitis” that includes Powassan virus disease and “Other Specified Rickettsiosis” and “Rickettsiosis, unspecified (includes rickettsial infection)” which are the closest available groups to anaplasmosis in the ICD-10-CA. For simplicity, the latter two groups were consolidated into one group hereafter referred to as “rickettsioses”. The information on the selected TBDs of interest was then extracted from the DAD using the respective ICD-10-CA codes (Table 1). A hospitalized case, is an inpatient, defined as an individual who has been admitted into the health care organization for medical and/or health services on a time limited basis. This is represented as an entry in the DAD database with at least one of the selected ICD-10-CA TBD codes. To better estimate the incidence of patients diagnosed with TBDs, only the first hospitalization within a one-year time frame was considered in the analysis and any subsequent hospitalizations within the same year were considered as duplicates and were excluded from the analysis as per other studies using similar data [22–24].

**Table 1:** Hospitalized patients diagnosed with tick-borne-disease (s), by disease category (International Classification of Diseases Codes, 10th Revision), from the Discharge Abstract Database, Canada, 2009−2021.

Disease code	Description	No. (%)
A69.2	Lyme disease	1,457 (89.6)
A79.9; A79.8	Rickettsiosis*	57 (3.5)
A77.0	Spotted fever due to *Rickettsia rickettsii* (includes: Rocky Mountain spotted fever and Sao Paulo fever)	38 (2.3)
A21; A21.0; A21.1; A21.2; A21.3; A21.7; A21.8; A21.9	Tularemia**	34 (2.1)
B60.0	Babesiosis	14 (0.9)
A84.4	Other tick-borne viral encephalitis (includes Powassan virus)	10 (0.6)
A68.1	Tick-borne relapsing fever (includes Relapsing fever due to any *Borrelia* species other than *Borrelia recurrentis*)	8 (0.5)
A93.2	Colorado tick fever	1 (0.1)
	**Multiple Diagnoses**	
A69.2; B60.0	Lyme disease + Babesiosis	2 (0.1)
A69.2; A68.1	Lyme disease + Tick-borne relapsing fever	1 (0.1)
A69.2; A79.9; A79.8	Lyme disease + Rickettsiosis*	1 (0.1)
A69.2; A77.0	Lyme disease + Spotted fever due to *Rickettsia rickettsii*	1 (0.1)
A79.9; A79.8; A68.1	Rickettsiosis* + Tick-borne relapsing fever	2 (0.1)
	Total	1,626 (100)

* Several ICD-10-CA codes were used to identify rickettsiosis; codes for rickettsiosis include “rickettsiosis unspecified” and “other specified rickettsiosis”.

** ICD-10-CA codes for tularemia include tularemia, ulceroglandular tularemia, oculoglandular tularemia, pulmonary tularemia, gastrointestinal tularemia, generalized tularemia, other forms of tularemia and tularemia, unspecified, some of which are likely transmitted by routes other than tick-borne.

It is possible that the reporting of hospitalizations and cases varied over time associated with impacts on public health and healthcare capacity during the COVID-19 pandemic. To explore this possibility, the numbers of LD hospitalizations reported in the DAD were compared across years as percentages of the numbers of cases reported through surveillance (collected through the LDES and the CNDSS systems, which together form the national surveillance system).

Descriptive analysis was conducted for hospitalized patients diagnosed with TBDs according to the year and month of admission to hospital, demographic characteristics (patient’s age and sex), and region of hospitalization (West Coast [British Columbia], Prairie provinces [Alberta, Saskatchewan, Manitoba], Central Canada [Ontario, Québec], Atlantic provinces [New Brunswick, Nova Scotia, Prince Edward Island and Newfoundland and Labrador]), and North (Yukon Territory, Northwest Territories and Nunavut). Due to the unavailability of data from Québec in the DAD databases, Central Canada refers only to Ontario in this study.

For the analysis of demographic and seasonal patterns, TBDs were categorized based on the vector tick species, into pathogens transmitted by *I*. *scapularis* and *I*. *pacificus* (causing rickettsiosis, tick-borne viral encephalitis and babesiosis) and pathogens transmitted by other tick species (causing tularemia, RMSF, tick-borne relapsing fever and Colorado tick fever).

Incidence rates were calculated using the respective census population estimates data from Statistics Canada as the denominator [25]. Changes in annual hospitalization incidence were visualised with 95% confidence intervals that used the population of Canada as the denominator. Data extraction and analysis were performed using SAS Enterprise Guide (EG) version 7.15 (SAS Institute Inc., USA).

## Results

From 2009 through 2021, a total of 1,626 unique patients, diagnosed with TBDs during their hospital stay, were identified in the DAD. The majority (99.6%; 1,619/1,626) had one TBD diagnosis including LD (n = 1,457) and other TBDs (n = 162), while seven patients were diagnosed with more than one TBD (Table 1).

### Annual trends

**Fig 1:**
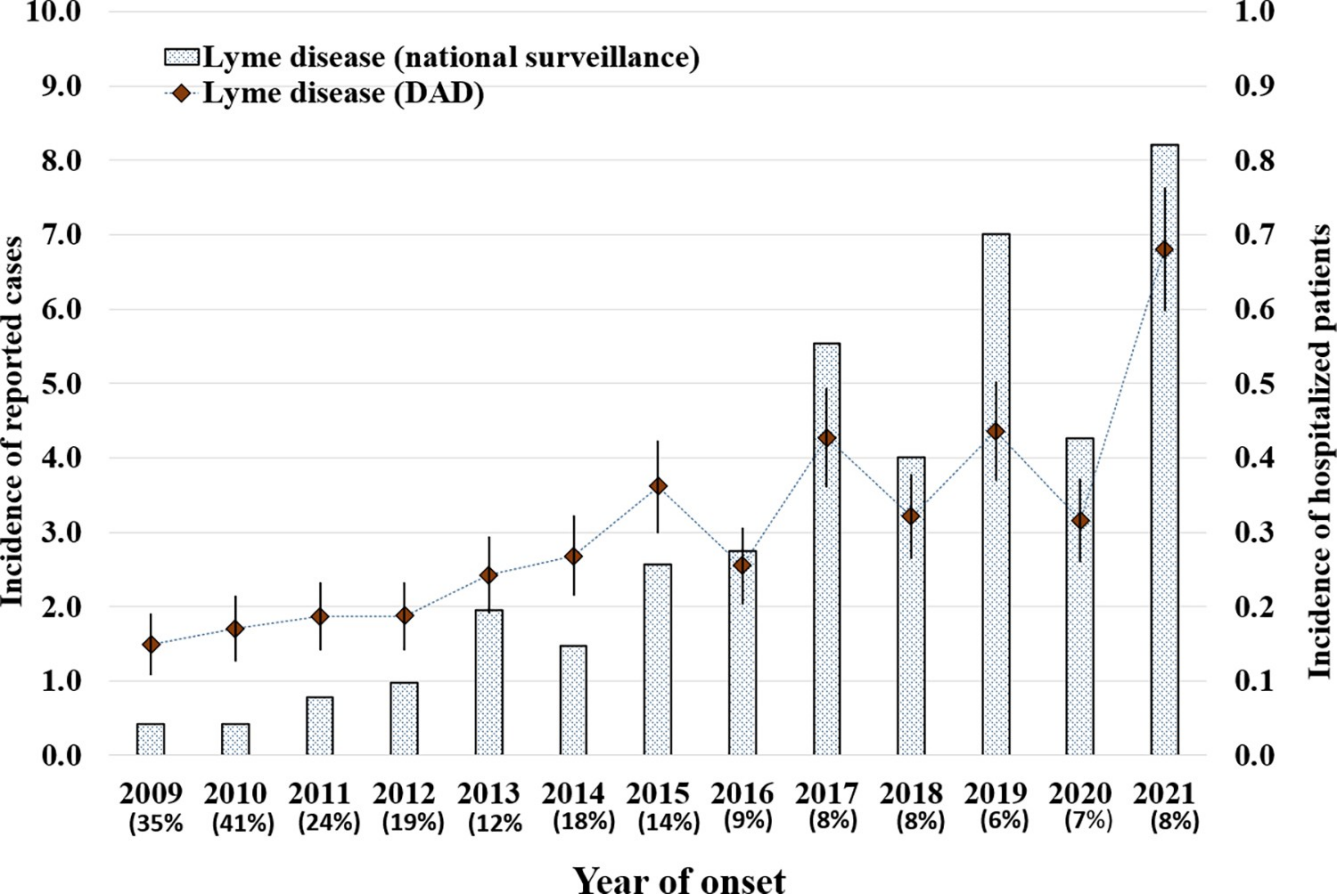
Annual incidence per 100,000 population of Lyme disease diagnosed patients in Discharge Abstract Database (DAD) and Lyme disease cases in national surveillance data, Canada, 2009−2021. The ratio of hospitalized patients with a diagnosis of Lyme disease to the number of cases reported in national surveillance data is shown as a percentage.

A total of 1,457 hospitalized patients were diagnosed with LD during the study period. Annual hospitalization counts for LD showed a significant increase from 2009 to 2021, rising from 50 to 259 (a five-fold increase). Annual incidence (per 100,000 population) for LD hospitalizations also increased steadily over the same period, with a more marked increase, with interannual variations, from 2017 to 2021. During this period incidence was highest in 2021 (0.7; 95% CI: 0.6−0.8) (Fig 1).

The annual number of LD diagnosed patients in DAD expressed as a percentage of the number of LD cases reported in national surveillance data steadily decreased from 35% in 2009 to 14% in 2015, and then decreased to between 6% and 9% during the period 2016 to 2021 (Fig 1).

**Fig 2:**
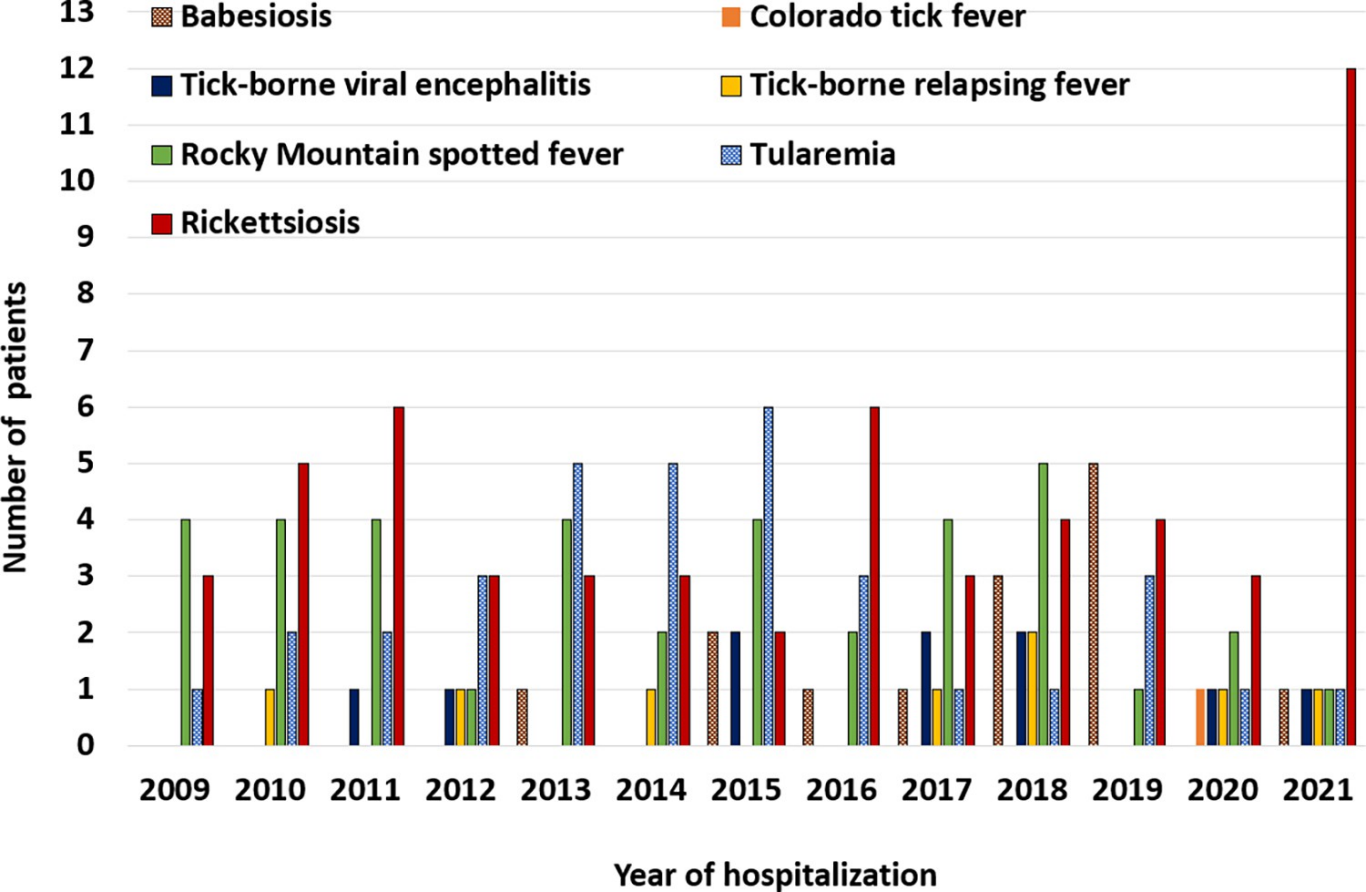
Annual tick-borne diseases hospitalizations, other than Lyme disease, from the Discharge Abstract Database using the International Classification of Diseases Codes, 10th Revision (ICD-10-CA), Canada, 2009−2021.

For the patients diagnosed with other tick-borne diseases (i.e. non-LD, n = 162); the annual patient counts with rickettsiosis (32.7% of patients), babesiosis (8.6% of patients) and tick-borne viral encephalitis (6.2% of patients) varied throughout the study period, with an almost 5-fold increase overall (from 3 to 14 patients) during the same period primarily due to the increase in patients with rickettsiosis (from 3 to 12 patients) (Fig 2), while for the remaining diagnoses, RMSF (23.5%), tularemia (21.0%), tick-borne relapsing fever (4.9%), and Colorado tick fever (0.6%), annual counts varied but overall the trend remained stable throughout the study period (Fig 2).

### Seasonal patterns

**Fig 3:**
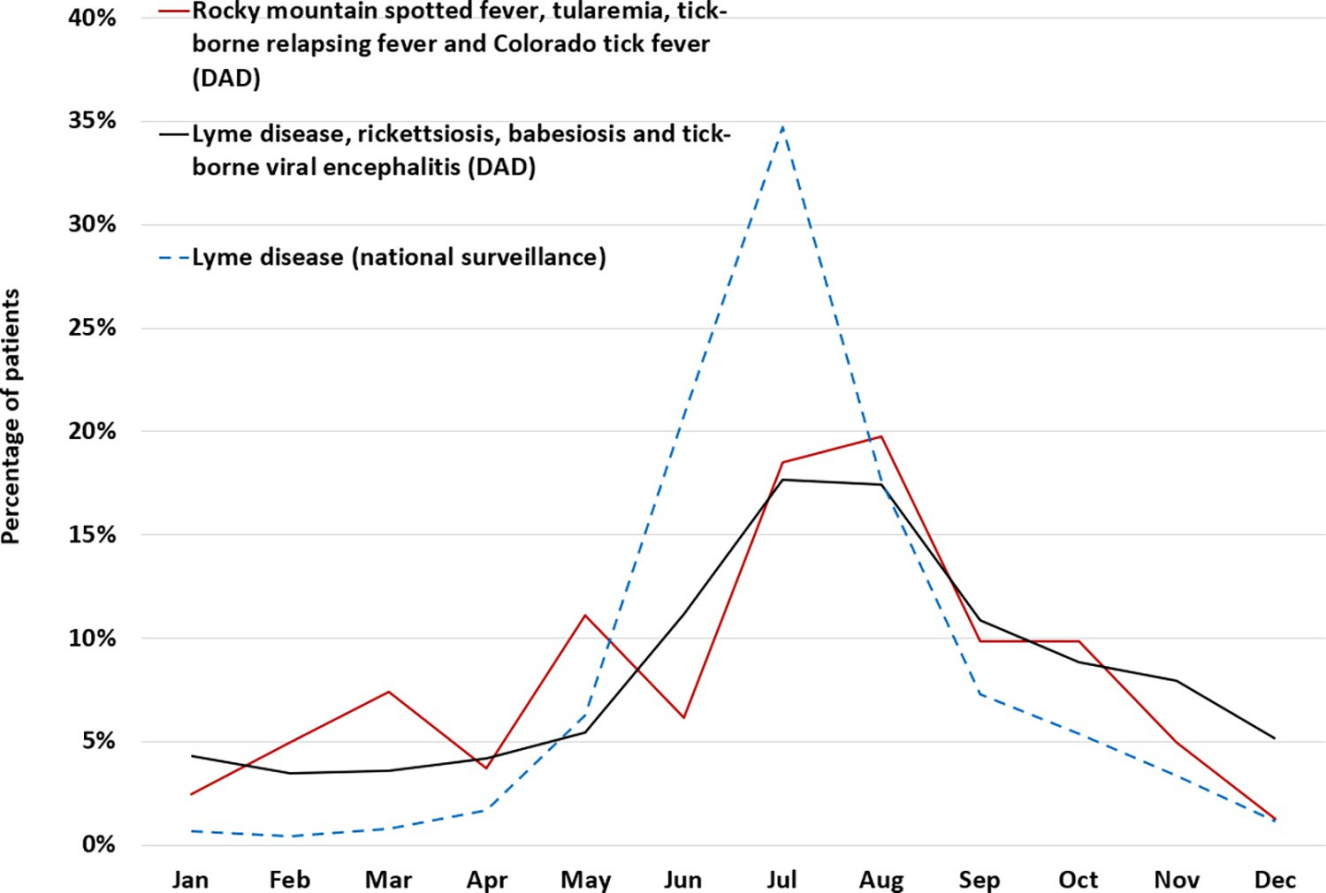
Seasonal distribution of Lyme disease cases reported in national surveillance data and other tick-borne disease hospitalizations (dates of onset or admission), from the Discharge Abstract Database (DAD) using the International Classification of Diseases Codes, 10th Revision (ICD-10-CA), Canada, 2009−2021. The percentage of patients was calculated by dividing the monthly patient count by the total number of patients.

Diagnosis of patients with TBDs during their hospital stay occurred throughout the year, but with a clear seasonal pattern. Patients diagnosed in the summer months of July and August accounted for 34.1% for the total of LD, rickettsiosis, babesiosis and tick-borne viral encephalitis; and for 38.3% for the total of RMSF, tularemia, tick-borne relapsing fever and Colorado tick fever (Fig 3).

### Geographic distribution

**Fig 4:**
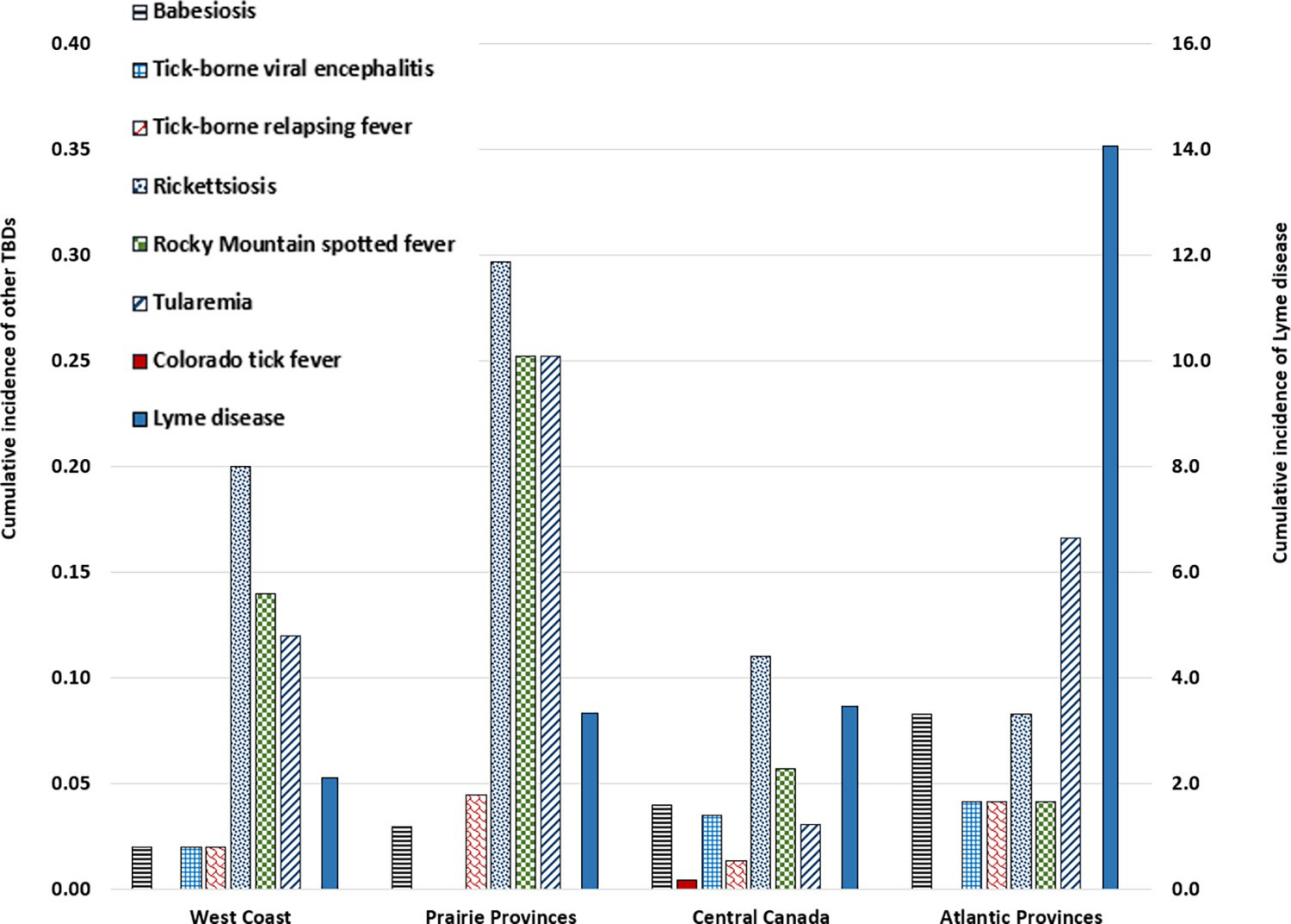
Regional distribution of incidence of hospitalizations (per 100,000 population) with diagnoses of tick-borne diseases, in data from the Discharge Abstract Database using the International Classification of Diseases Codes, 10th Revision (ICD-10-CA), Canada, 2009−2021.

Among the hospitalizations with a diagnosis of LD (n = 1,457), the cumulative incidence rates were highest in the Atlantic provinces (14.1 per 100,000 population) followed by Central Canada (3.5), the Prairie provinces (3.3), and the West Coast (2.1) (Fig 4). Central Canada accounted for the majority of hospitalizations (53.9%; n = 786), followed by the Atlantic region (23.2%; n = 339), the Prairie provinces (15.4%; n = 225), and the West Coast (7.2%; n = 106). Only one LD-coded hospitalization was documented in one of the Territories.

Among the 162 hospitalized patients with other TBDs diagnoses, the cumulative incidence rates for rickettsiosis (0.3 and 0.2) and RMSF (0.3 and 0.1) were highest in the Prairie provinces, followed by the West Coast. Most of the rickettsiosis were recorded in Central Canada (25/57) and the Prairie provinces (20/57). Rocky Mountain spotted fever was recorded most frequently in the Prairie provinces (17/38) followed by Central Canada (13/38).

The Prairie provinces had the highest cumulative incidence rate of tularemia (0.3) and half of the total number of hospitalizations (17/34). The cumulative incidence rate of tick-borne viral encephalitis was highest and similar in Central Canada and the Atlantic provinces (0.04) with most hospitalizations recorded in Central Canada (8/10). Cumulative incidence for babesiosis was highest in the Atlantic provinces (0.08) followed by Central Canada (0.04) with the majority (9/14) recorded in Central Canada. Incidence rate of tick-borne relapsing fever was highest and similar in the Prairie and Atlantic provinces (0.04), with most hospitalizations (6/8) recorded in the Prairie provinces and Central Canada (Fig 4).

### Demographic characteristics

**Fig 5:**
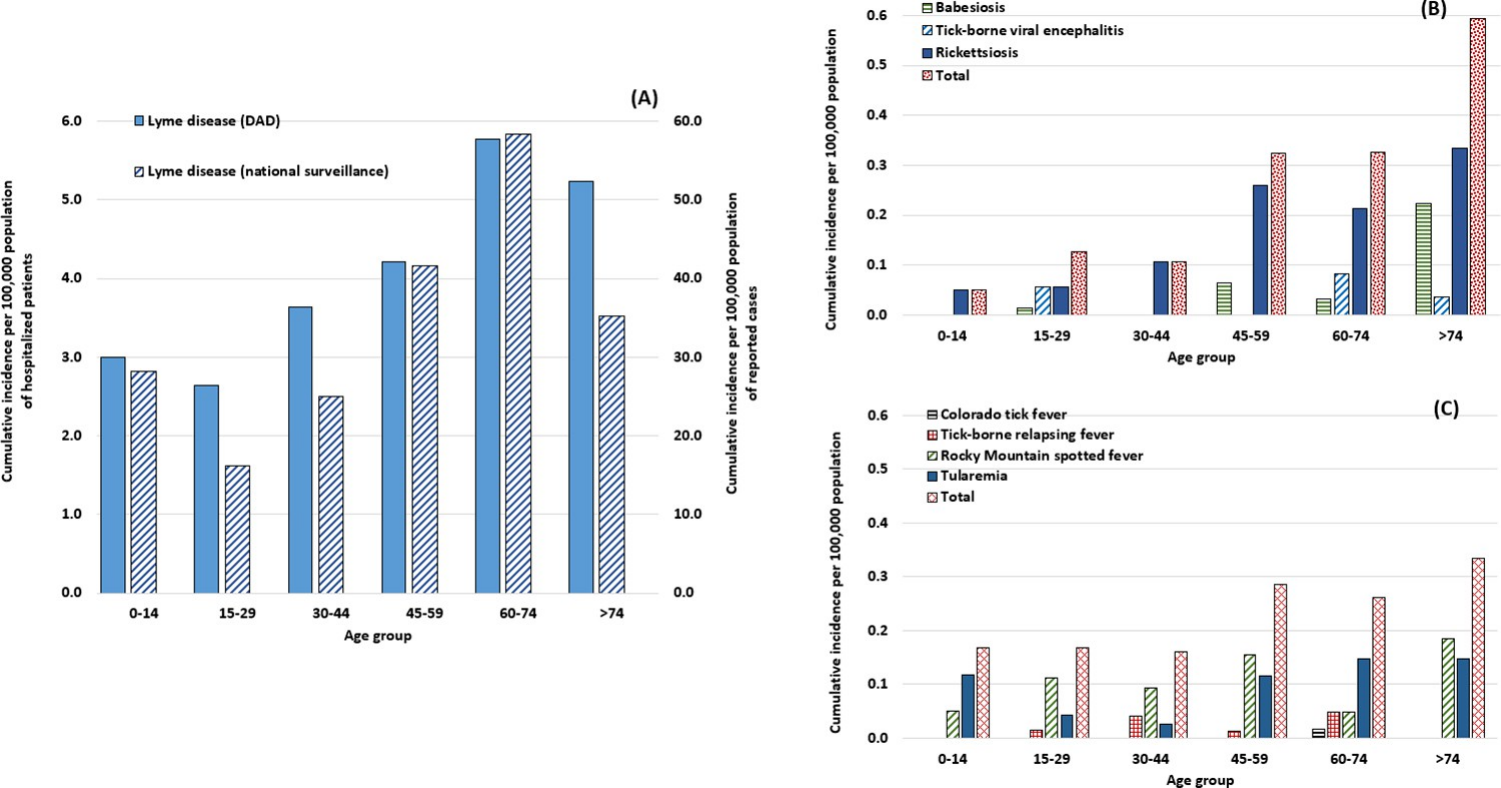
Cumulative incidence rates of hospitalizations related to tick-borne diseases, by age group, Canada, 2009−2021. Graph A shows cumulative incidence for Lyme disease hospitalizations with cumulative incidence of cases reported in national surveillance data. Graph B presents hospitalizations related to non-Lyme disease *Ixodes scapularis/pacificus*-borne diseases (babesiosis, tick-borne viral encephalitis and rickettsiosis). Graph C presents hospitalizations related to the other tick-borne diseases transmitted by other vectors (Colorado tick fever, tick-borne relapsing fever, RMSF, and tularemia). Hospitalization data from the Discharge Abstract Database (DAD) using the International Classification of Diseases Codes, 10th Revision (ICD-10-CA).

The age distribution of patients hospitalized with a diagnosis of LD showed the highest cumulative incidence (5.8 per 100,000) in the 60–74 age group, which accounted for 23.6% of overall patients (n = 1,457) (Fig 5A). For the group of diseases that include rickettsiosis, babesiosis and tick-borne viral encephalitis, the cumulative incidence was highest in the ≥74 age group (0.6) and accounted for 38.8% of overall patients (n = 81) (Fig 5B).

The cumulative incidence of RMSF, tularemia, tick-borne relapsing fever and Colorado tick fever combined (n = 81) was also higher in the ≥74 age group (0.3) accounting for 24.2% of the total for these patients (Fig 5C).

The proportion of LD cases in male patients (50.6%; 738/1,457) and female patients was similar, while for the combined group of *I*. *scapularis/I*. *pacificus*-transmitted pathogens (causing rickettsiosis, babesiosis and tick-borne viral encephalitis), and for pathogens transmitted by other tick species (causing RMSF, tularemia, tick-borne relapsing fever and Colorado tick fever), the proportion of cases that were males was higher than females, 66.6%; (54/81) and 61.7% (50/81), respectively.

## Discussion

This study presents the results of a retrospective analysis of thirteen years of Discharge Abstract Database (DAD) on diagnosed patients with TBDs in acute care hospitals in Canada.

From 2009 through 2021, 1,457 patients were diagnosed with LD during their hospital stay in Canada. Results from the national surveillance data showed an important shift in the annual trend of reported cases starting in 2015, with an almost doubling of cases compared to previous years [26]. Hence, we estimate that from 2015 through 2021, the annual incidence was 0.4 per 100,000 (CI 95%; 0.3–0.5) and 149 (CI 95%; 125−172) patients were diagnosed with LD each year during their stay in acute care facilities in Canada, which accounts for 9% of the overall cases reported through the national surveillance data during this period.

The annual incidence of hospitalized patients with LD showed interannual variation and a marked increase over the study period, which was consistent with cases reported in the national surveillance data [26, 27].

Comparison between the DAD and national surveillance data (percentage calculated by dividing the number of hospitalized patients by the number of reported cases through routine surveillance) showed a steady decrease in annual percentage from 35% in 2009 to between 6% and 9% during the period 2016 to 2021 (Fig 1), which suggests likely improvements in detection, treatment and reporting of early LD by general practitioners. Declines in both hospitalizations and reported cases occurred in 2020, which could reflect an impact of the COVID-19 pandemic on both recording and reporting of LD cases in Canada [28]. In 2021, both recorded hospitalizations and reported cases reached the highest incidence of the study, which may be consistent with the ongoing emergence of LD in Canada [29].

Misdiagnosis is possible as LD has a broad spectrum of manifestations that overlap with other diseases [30], and it is possible that a disease code recorded in the discharge abstract does not always indicate that was the actual cause of hospitalization [31].

Despite these limitations, the epidemiologic patterns of LD diagnoses in hospital settings are consistent with those reported through the national surveillance system.

A clear seasonal pattern is observed as well, with most admissions having occurred in the summer months of July and August. Some admissions in the fall and winter are likely associated with disseminated LD such as neurologic disorders or Lyme arthritis that can manifest weeks to months after an untreated infection. The age distribution for patients diagnosed with LD aligns with the incidences obtained through national surveillance data, with the highest proportion observed in adults aged 60−74 years [27].

The finding of similar proportions of male and female patients being diagnosed with LD is reported in a study conducted in a LD-endemic area in the USA [32]; this contrasts with a greater proportion of male cases reported through the national surveillance system.

The majority of patients diagnosed with LD were residents of areas at risk of LD where the main vectors of LD, the *I*. *scapularis* and *I*. *pacificus*, ticks are endemic [26]. The finding of patients diagnosed with LD during their hospital stay in the Prairie provinces of Saskatchewan and Alberta is likely due to exposure to LD-endemic areas within Canada or elsewhere, primarily the USA [33, 34], as to date no LD at-risk areas have been identified in this region. This further underscores the importance of public health actions across Canada to enhance the knowledge of health practitioners and the general public on the importance of timely detection and early treatment of LD in order to minimize the burden of hospitalizations even though there are no known within-province risk areas in this region [35].

During the study period, an estimated of 162 patients were diagnosed with TBDs other than LD, including 81 patients with *I*. *scapularis/pacificus*-borne pathogens (rickettsiosis, babesiosis and tick-borne viral encephalitis) and 81 patients diagnosed with other tick species-borne pathogens (tularemia, RMSF, tick-borne relapsing fever and Colorado tick fever). Cases diagnosed with *I*. *scapularis/pacificus*-borne pathogens increased over the study period primarily due to an increase in cases of rickettsiosis, with annual case counts almost doubling during this period. For the other tick species transmitted pathogens, the annual case counts varied but didn’t show any constant increase over the study period.

Epidemiologic features of the non-LD *I*. *scapularis/pacificus*-borne pathogens show some contrast with LD. The age group most frequently hospitalized due to non-LD *I*. *scapularis/pacificus*-borne pathogens was the ≥74 years old, while for LD younger patients were more likely to be hospitalized and be reported through the national surveillance system as well as in this study. Further studies are needed to understand these differences.

Hospital admissions of the other tick-borne diseases show a similar seasonal pattern to those of LD, with most admissions during the months when ticks are most active. As for LD, admissions in fall and winter could correspond to complications of untreated disease or the occurrence of severe manifestations as the disease progresses. *Francisella tularensis* can be transmitted year-around through various routes. Apart from transmission via tick or fly bites, it can also be spread through inhalation of contaminated dust, consumption of contaminated food or water, or contact with infected animals [36]. In addition to these alternative transmission routes, changes in tick habitat due to climate change and off-peak season travel to endemic areas could result in a weaker observed pattern of seasonal transmission compared to LD.

The cumulative incidence rate of the non-RMSF rickettsiosis hospitalizations was higher in the Prairies followed by the West Coast which aligns with the geographic distribution of the *Dermacentor andersoni* (*D*. *andersoni*) and *D*. *variabilis* ticks, vectors of rickettsiosis in these regions. While *I*. *scapularis* is the primary vector of *A*. *phagocytophilum* in Eastern Canada, the lack of DAD data from Québec could have led to a considerable underestimation of the number of hospitalizations due to infections with *A*. *phagocytophilum*. In 2021, an unusual cluster of 22 cases of human granulocytic anaplasmosis had been reported in Québec [4] likely associated with high prevalence of *A*. *phagocytophilum* infection in ticks in the region. However, it is possible that some RMSF diagnoses were classified as rickettsiosis.

The highest cumulative incidence rates of hospitalizations with diagnoses of RMSF and tularemia occurred in the Prairies, followed by the West Coast for RMSF, and the Atlantic provinces for tularemia. This regional distribution aligns with the geographic distribution of *D*. *andersoni* (main vector of *Rickettsia rickettsii* agent of RMSF) [37, 38] found in southwestern Saskatchewan and the southern half of Alberta and British Columbia and *D*. *variabilis* (main vector of *Francisella tularensis* agent of tularemia) [39–41] which is endemic to much of southern Canada [5, 39–43]. In addition, tularemia cases have been reported from most provinces and territories in the last years [44] which corroborate these findings.

The annual incidence of hospitalized patients diagnosed with babesiosis showed a modest upward trend until 2019, with no additional diagnoses being recorded thereafter. A survey of blood donors found evidence that human transmission of *B*. *microti* occurs across Canada [13, 15], and field tick surveillance found that the protozoan is present in most of the provinces where *I*. *scapularis* and *I*. *pacificus* occur. The low incidence of babesiosis hospitalizations should be interpreted with caution, as severe manifestations of babesiosis that require hospitalization are limited to individuals with underlying health conditions or the elderly [45].

The highest cumulative incidence rates of tick-borne viral encephalitis’ hospitalizations occurred in Ontario and the Atlantic provinces. In North America, the tick-borne flavivirus known to cause tick-borne viral encephalitis in humans are Powassan viruses (Lineage I and lineage II [46]). It is very possible these are locally acquired infections as the tick vectors (*I*. *cookie* for lineage 1 and *I*. *scapularis* for lineage 2) are endemic to these regions [14, 15, 47].

The most common cause of tick-borne relapsing fever in North America is *B*. *hermsii*, which is endemic to southeastern British Columbia and where human infections have previously been reported [6]. Our finding that most hospitalizations due to tick-borne relapsing fever were from the Prairies and Atlantic provinces is likely consistent with exposure to the known geographic distribution of *B*. *hermsii* transmission cycles in British Columbia [6], northwestern USA [48] or elsewhere.

The single diagnosis of Colorado tick fever recorded in Ontario is likely an infection acquired during travel to an endemic area in the USA or Canadian Prairies provinces where the tick vector, *D*. *andersoni*, is endemic [38, 41].

The finding that multiple diagnoses were documented in 0.4% of all diagnoses during the same patient’s hospitalization stay suggests that co-infections, albeit rare, can occur in Canada. *Ixodes scapularis* ticks carrying multiple pathogens and co-infections in humans have been documented, both locally [14] and in neighbouring states of the USA [49]. Co-infections can exacerbate the manifestations of LD and other TBDs and vice versa [50, 51], and often have overlapping symptoms which make diagnosis with multiple pathogens challenging [52], particularly in areas where novel pathogens are emerging.

There were some limitations to the present study. First, the incidence of tick-borne disease hospitalizations are likely underestimated since data from Québec were not available. Hospitalization in this study cannot necessarily be attributed to TBD infection or the primary reason for the patient’s admission to hospital; it indicates, rather, that the patient received a diagnosis of one or more TBDs during their hospital stay.

## Conclusions

This is the first retrospective analysis that provides insights into hospitalization data for LD and other TBDs in Canada over a thirteen years timeframe.

The incidence of patients who received a diagnosis of LD during their hospital stay increased over the study period as did the number of cases reported through the national surveillance system. Despite low case number for the other TBDs, our findings of similar age, seasonality, and geographic distribution for LD cases in the hospitalization data (DAD) system and the national surveillance system suggests the reliability of the DAD information to support monitoring and detection of emerging TBDs in Canada.

In conclusion, monitoring hospitalization data can provide insight into the burden of emerging TBDs, the severity of illness and the population groups most at risk.

However, the lack of specific codes for emerging TBDs such as anaplasmosis and Powassan virus disease pose a notable limitation to the accuracy of estimates. Hence, enhancement of the ICD-10-CA codes list would be useful in anticipation of the emergence of TBDs in Canada.

## References

[1] Bouchard C, Dibernardo A, Koffi JK, Wood H, Leighton PA, Lindsay LR. Increased risk of tick-borne diseases with climate change. Can Commun Dis Rep. 2019;45:4. 10.14745/ccdr.v45i04a02 PMC6587693 31285697

[2] Gasmi S, Bouchard C, Ogden NH, Adam-Poupart A, Pelcat Y, Rees E, et al. Evidence for increasing densities and geographic ranges of tick species of public health significance other than *Ixodes scapularis* in Québec, Canada. PLoS One. 2018;13(8):e0201924. 30133502 10.1371/journal.pone.0201924 PMC6104943

[3] Duplaix L, Wagner V, Gasmi S, Lindsay LR, Dibernardo A, Thivierge K, et al. Exposure to Tick-Borne Pathogens in Cats and Dogs Infested With Ixodes scapularis in Quebec: An 8-Year Surveillance Study. Front Vet Sci. 2021;8. doi: 10.3389/fvets.2021.696815 34336980 PMC8321249

[4] Campeau L, Roy V, Petit G, Baron G, Blouin J, Carignan A. Identification of an unusual cluster of human granulocytic anaplasmosis in the Estrie region, Québec, Canada in 2021. Can Commun Dis Rep. 2022;48(5):188–95. 38090114 10.14745/ccdr.v48i05a02 PMC10712899

[5] Ogden NH, Bouchard C, Brankston G, Brown E, Corrin T, Dibernardo A, Drebot MA. Infectious diseases. In: Berry P, Schnitter R, editors. Health of Canadians in a Changing Climate: Advancing Our Knowledge for Action, 2022 https://changingclimateca/health-in-a-changing-climate/. Government of Canada.

[6] Morshed M, Drews SJ, Lee M-K, Fernando K, Man S, Mak S, et al. Tick-borne relapsing fever in British Columbia: a 10-year review (2006–2015). BC Med J. 2017;59(8):412–7.

[7] Hughes HR, Velez JO, Fitzpatrick K, Davis EH, Russell BJ, Lambert AJ, et al. Genomic Evaluation of the Genus Coltivirus Indicates Genetic Diversity among Colorado Tick Fever Virus Strains and Demarcation of a New Species. Diseases. 2021;9(4):92. doi: 10.3390/diseases9040092 34940030 PMC8700517

[8] Satia I, Cusack R, Greene JM, O’Byrne PM, Killian KJ, Johnston N. Prevalence and contribution of respiratory viruses in the community to rates of emergency department visits and hospitalizations with respiratory tract infections, chronic obstructive pulmonary disease and asthma. PLoS One. 2020;15(2):e0228544. doi: 10.1371/journal.pone.0228544 32027687 PMC7004370

[9] Joseph K, Liu S, Rouleau J, Kirby RS, Kramer MS, Sauve R, et al. Severe maternal morbidity in Canada, 2003 to 2007: surveillance using routine hospitalization data and ICD-10CA codes. J Obstet Gynaecol Can. 2010;32(9):837–46. doi: 10.1016/S1701-2163(16)34655-2 21050516

[10] Kramer MS, Rouleau J, Baskett TF, Joseph K. Amniotic-fluid embolism and medical induction of labour: a retrospective, population-based cohort study. The Lancet. 2006;368(9545):1444–8. doi: 10.1016/S0140-6736(06)69607-4 17055946

[11] Parkins MD, Church DL, Jiang XY, Gregson DB. Human granulocytic anaplasmosis: First reported case in Canada. Can J Infect Dis Med Microbiol. 2009;20(3):e100–e2. doi: 10.1155/2009/124173 20808448 PMC2770309

[12] Scott JD. First record of locally acquired human babesiosis in Canada caused by Babesia duncani: A case report. SAGE Open Med Case Rep. 2017;5:2050313X17725645. doi: 10.1177/2050313X17725645 28890784 PMC5580841

[13] Tonnetti L, O’Brien SF, Grégoire Y, Proctor MC Drews SJ, Delage G, et al. Prevalence of Babesia in Canadian blood donors: June–October 2018. Transfusion. 2019;59(10):3171–6. doi: 10.1111/trf.15470 31385317

[14] Wilson CH, Gasmi S, Bourgeois A-C, Badcock J, Chahil N, Kulkarni MA, et al. Surveillance for Ixodes scapularis and Ixodes pacificus ticks and their associated pathogens in Canada, 2019. Can Commun Dis Rep. 2022;48(5):208. doi: 10.14745/ccdr.v48i05a04 37325256 PMC10262936

[15] Guillot C, Badcock J, Clow K, Cram J, Dergousoff S, Dibernardo A, et al. Canadian Lyme Sentinel surveillance report, 2019. Can Commun Dis Rep. 2020;46(10). 10.14745/ccdr.v46i10a08 PMC7723316 33315999

[16] Nelder MP, Russell CB, Dibernardo A, Clow KM, Johnson S, Cronin K, et al. Monitoring the patterns of submission and presence of tick-borne pathogens in *Ixodes scapularis* collected from humans and companion animals in Ontario, Canada (2011–2017). Parasites & vectors. 2021;14(1):1–13. 34001256 10.1186/s13071-021-04750-1 PMC8127263

[17] Nelder MP, Russell CB, Sheehan NJ, Sander B, Moore S, Li Y, et al. Human pathogens associated with the blacklegged tick *Ixodes scapularis*: a systematic review. Parasites & vectors. 2016;9(1):265. doi: 10.1186/s13071-016-1529-y 27151067 PMC4857413

[18] Smith K, Oesterle PT, Jardine CM, Dibernardo A, Huynh C, Lindsay R, et al. Powassan virus and other arthropod-borne viruses in wildlife and ticks in Ontario, Canada. Am J Trop Med Hyg. 2018;99(2):458. doi: 10.4269/ajtmh.18-0098 29869604 PMC6090327

[19] Centers for Disease Control and Prevention. Tickborne Disease Surveillance Data Summary. 2022. https://www.cdc.gov/ticks/data-summary/index.html.

[20] Ogden NH, Lindsay LR, Hanincova K, Barker IK, Bigras-Poulin M, Charron DF, et al. Role of migratory birds in introduction and range expansion of *Ixodes scapularis* ticks, and *Borrelia burgdorferi* and *Anaplasma phagocytophilum* in Canada. Appl Environ Microbiol. 2008;74(6):1780–90. 18245258 10.1128/AEM.01982-07 PMC2268299

[21] Ogden NH, Artsob H, Margos G, Tsao J, Sonenshine D, Roe M. Non-rickettsial tick-borne bacteria and the diseases they cause. Biology of ticks. 2011;2:278–312.

[22] Nelson CA, Saha S, Kugeler KJ, Delorey MJ, Shankar MB, Hinckley AF, Mead PS. Incidence of clinician-diagnosed Lyme disease, United States, 2005–2010. Emerg Infect Dis. 2015;21(9):1625. doi: 10.3201/eid2109.150417 26291194 PMC4550147

[23] Stanek G, Wormser GP, Gray J, Strle F. Lyme borreliosis. The Lancet. 2012;379(9814):461–73. 10.1016/S0140-6736(11)60103-7 21903253

[24] Steere AC, Strle F, Wormser GP, Hu LT, Branda JA, Hovius JWR, et al. Lyme borreliosis. Nat Rev Dis Primers. 2016;2:16090. doi: 10.1038/nrdp.2016.90 27976670 PMC5539539

[25] Statistics Canada. Population estimates on July 1st, by age and sex. 2022. https://www150.statcan.gc.ca/t1/tbl1/en/cv.action?pid=1710000501#timeframe.

[26] Government of Canada. Lyme disease: Surveillance. 2023. https://www.canada.ca/en/public-health/services/diseases/lyme-disease/surveillance-lyme-disease.html#a2.

[27] Gasmi S, Koffi J, Nelder M, Russell C, Graham-Derham S, Lachance L, et al. Surveillance for Lyme disease in Canada, 2009–2019. Can Commun Dis Rep. 2022;48(5):219–27. doi: 10.14745/ccdr.v48i05a05 38105769 PMC10723632

[28] McCormick DW, Kugeler KJ, Marx GE, Jayanthi P, Dietz S, Mead P, et al. Effects of COVID-19 pandemic on reported Lyme disease, United States, 2020. Emerg Infect Dis. 2021;27(10):2715. doi: 10.3201/eid2710.210903 34545801 PMC8462321

[29] Ogden NH, Dumas A, Gachon P, Rafferty E. Estimating the Incidence and Economic Cost of Lyme Disease Cases in Canada in the 21st Century with Projected Climate Change. Environ Health Perspect. 2024;132(2):027005. doi: 10.1289/EHP13759 38349724 PMC10863724

[30] Foster ZJ, Day AL, Miller J. Polyarticular Joint Pain in Adults: Evaluation and Differential Diagnosis. Am Fam Physician. 2023;107(1):42–51. 36689970

[31] Rutz H, Hogan B, Hook S, Hinckley A, Feldman K. Exploring an alternative approach to Lyme disease surveillance in Maryland. Zoonoses Public Health. 2018;65(2):254–9. doi: 10.1111/zph.12446 29411541 PMC10880063

[32] Kwit NA, Nelson CA, Max R, Mead PS, editors. Risk factors for clinician-diagnosed Lyme arthritis, facial palsy, carditis, and meningitis in patients from high-incidence states. Open Forum Infect Dis; 2018: Oxford University Press US. 10.1093/ofid/ofx254 PMC5757643 29326960

[33] Government of Canada. Lyme disease surveillance report: Annual edition 2018. 2021. https://www.canada.ca/en/public-health/services/publications/diseases-conditions/lyme-disease-surveillance-report-2018.html.

[34] Government of Canada. Lyme disease surveillance report: Annual edition 2019. 2022. https://www.canada.ca/en/public-health/services/publications/diseases-conditions/lyme-disease-surveillance-report-2019.html.

[35] Gasmi S, Ogden NH, Leighton PA, Adam-Poupart A, Milord F, Lindsay LR, et al. Practices of Lyme disease diagnosis and treatment by general practitioners in Quebec, 2008–2015. BMC Fam Pract. 2017;18(1):65. doi: 10.1186/s12875-017-0636-y 28532428 PMC5441092

[36] Pal M, Shuramo MY, Gutama KP. Tularaemia: A Re-emerging infectious zoonotic disease of public health significance. Int J Clin Exp Med Res. 2022;6:48–51.

[37] University of Saskatchewan. *Dermacentor andersoni*: the Rocky Mountain wood tick. 2021. https://wcvm.usask.ca/learnaboutparasites/parasites/dermacentor-andersoni-the-rocky-mountain-wood-tick.php.

[38] Lysyk TJ, Dergousoff SJ, Rochon K, Chilton NB, Smith AM. Distribution of Dermacentor andersoni (Acari: Ixodidae) in grassland regions of Alberta, Canada. J Med Entomol. 2021;58(4):1750–61. doi: 10.1093/jme/tjab019 33675646

[39] Wood H, Dillon L, Patel SN, Ralevski F. Prevalence of Rickettsia species in Dermacentor variabilis ticks from Ontario, Canada. Ticks Tick Borne Dis. 2016;7(5):1044–6. doi: 10.1016/j.ttbdis.2016.06.001 27318438

[40] Clow KM, Weese JS, Rousseau J, Jardine CM. Microbiota of field-collected *Ixodes scapularis* and *Dermacentor variabilis* from eastern and southern Ontario, Canada. Ticks Tick Borne Dis. 2018;9(2):235–44. doi: 10.1016/j.ttbdis.2017.09.009 29042239

[41] Gordon J, McLaughlin B, Nitiuthai S. Tularaemia transmitted by ticks (Dermacentor andersoni) in Saskatchewan. Can J Comp Med. 1983;47(4):408. 6667429 PMC1235967

[42] Martin T, Holmes IH, Wobeser GA, Anthony RF, Greefkes I. Tularemia in Canada with a focus on Saskatchewan. Can Med Assoc J. 1982;127(4):279. 7046896 PMC1861874

[43] Antonation K, Bekal S, Côté G, Dallaire A, Corbett C. Multiple‐locus variable‐number tandem‐repeat analysis of Francisella tularensis from Quebec, Canada. Lett Appl microbiology. 2015;60(4):328–33. doi: 10.1111/lam.12371 25442329

[44] Government of Canada. Reported cases from 1924 to 2020 in Canada—Notifiable diseases on-line. 2023. https://diseases.canada.ca/notifiable/charts?c=pl

[45] Centers for Disease Control and Prevention. Babesiosis. 2023. https://www.cdc.gov/parasites/babesiosis/gen_info/faqs.html.

[46] Dobler G. Zoonotic tick-borne flaviviruses. Vet Microbiol. 2010;140(3–4):221–8. doi: 10.1016/j.vetmic.2009.08.024 19765917

[47] Corrin T, Greig J, Harding S, Young I, Mascarenhas M, Waddell LA. Powassan virus, a scoping review of the global evidence. Zoonoses Public Health. 2018;65(6):595–624. doi: 10.1111/zph.12485 29911344

[48] Centers for Disease Control and Prevention. Tick and Loose-Borne Relapsing Fever. 2023. https://www.cdc.gov/relapsing-fever/index.html.

[49] Wormser GP, McKenna D, Scavarda C, Cooper D, El Khoury MY, Nowakowski J, et al. Co-infections in persons with early Lyme disease, New York, USA. Emerg Infect Dis. 2019;25(4):748. doi: 10.3201/eid2504.181509 30882316 PMC6433014

[50] Djokic V, Akoolo L, Primus S, Schlachter S, Kelly K, Bhanot P, et al. Protozoan parasite Babesia microti subverts adaptive immunity and enhances Lyme disease severity. Front Microbiol. 2019;10:1596. doi: 10.3389/fmicb.2019.01596 31354683 PMC6635642

[51] Westwood ML, Peters JL, Rooney TP. Prevalence and coinfection of three tick-borne pathogens in questing adult blacklegged ticks Ixodes scapularis (Vilas county, Wisconsin). Vector Borne Zoonotic Dis. 2020;20(8):633–5. doi: 10.1089/vbz.2020.2619 32283047

[52] Carson DA, Kopsco H, Gronemeyer P, Mateus-Pinilla N, Smith GS, Sandstrom EN, et al. Knowledge, attitudes, and practices of Illinois medical professionals related to ticks and tick-borne disease. One Health. 2022;15:100424. doi: 10.1016/j.onehlt.2022.100424 36277108 PMC9582564

